# THE ABCD FROM 1986 TO 2021: VICTORIOUS TRAJECTORY!

**DOI:** 10.1590/0102-672020210002e1609

**Published:** 2021-10-18

**Authors:** Osvaldo MALAFAIA

**Affiliations:** 1 CBCD Ex-President and ABCD Editor-in-Chief from 2001 to 2021

The ABCD - Brazilian Archives of Digestive Surgery, as can be seen in the “Presentation”^1^ in Figure 1, was conceived by Prof. Dr. Henrique Walter Pinotti, Professor of Digestive Surgery at the Faculty of Medicine, University of São Paulo, São Paulo, SP, Brazil and had its first issue published in 1986 final months. Therefore, in this year of 2021, with 35 years without interruptions, was edited four times a year. Its objective from the beginning was - and still is - to publicize the advances in gastrointestinal surgery in the country, as well as that of its related areas - nutrition, digestive endoscopy, experimental surgery, surgical technique, general surgery - and other aspects of gastroenterology in general.

**FIGURE 1:**
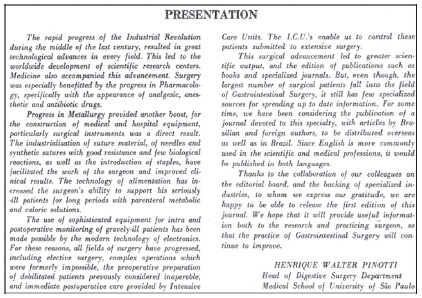
Original text in the presentation of the first issue of ABCD to the Brazilian scientific scenery

In the early years it was edited by Prof. Pinotti, assisted by the group of assistants and professors linked to him, among which can be cited Profs. Drs. Bruno Zilberstein, Ivan Cecconello and Joaquim José Gama Rodrigues. At the end of the 90’s, Prof. Pinotti transferred the journal to be edited by the Brazilian College of Digestive Surgery (CBCD) and, since then, it has become that way, thanks to the efforts of Profs. Drs. Bruno Zilberstein, Osvaldo Malafaia and, from 2010, also by Prof. Dr. Nelson Adami Andreollo, proposing new directions for the journal (Figure 2).

**FIGURE 2:**
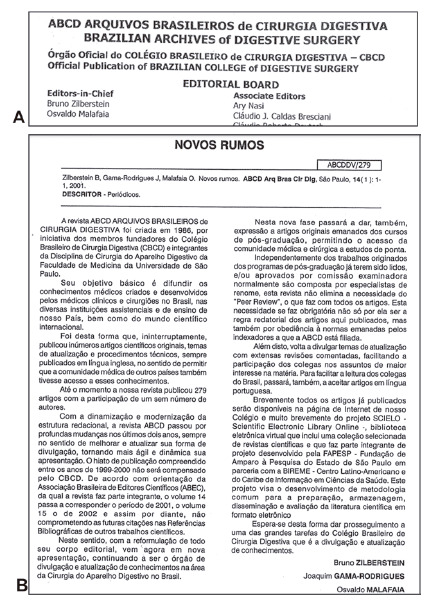
A) Part of the Editorial Board since 2001; B) article with purposes for ABCD in 2001

The first full article published in 1986 (Figure 3) was by the creator and mentor of the ABCD, Prof. Dr. Henrique Walter Pinotti talking about the surgeon-patient relationship^1^. Although the text was written 35 years ago, the concepts issued at the time are still very current. And in the presentation of this new journal he stated:

*“...due to the fact that most surgical patients are in the field of Gastrointestinal Surgery, there are few specialized sources to disseminate updated information. For some time now, we have been considering the publication of a Journal dedicated to this specialty, with articles by Brazilian and foreign authors, to be distributed abroad as well as in Brazil. Since English is more commonly used in medical and scientific specialties, it would be published in both languages”...*

**FIGURE 3:**
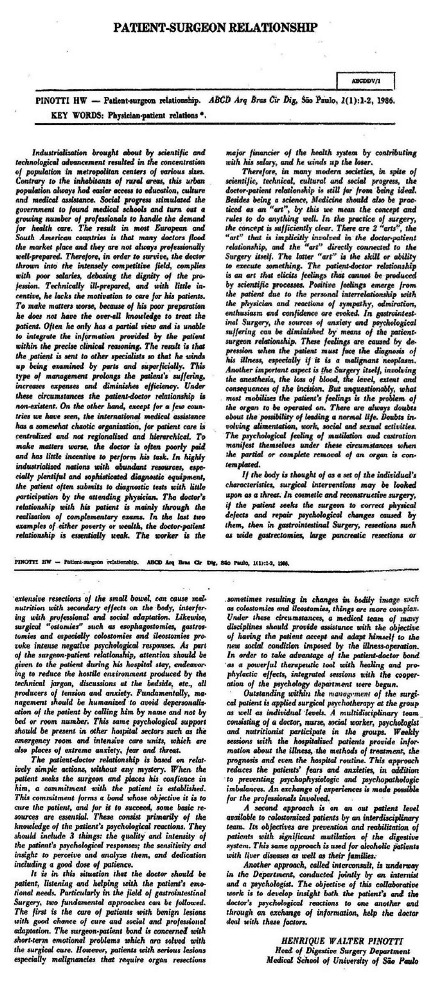
First ABCD scientific article and signed by Prof. Dr. Henrique Walter Pinotti

Prof. Pinotti in his speech during the beginning of the activities of the Brazilian College of Digestive Surgery (CBCD) in 1988 emphasized that:

*...”With education, not only information is provided, but high-level professionals are formed, who can develop their knowledge, who know how to correctly apply their resources and thus able to benefit their patients... We must in our College, in the area of education, to develop the spirit to each person who wants to teach can find many who want to learn”. And that the successes achieved in the safe training constitute stimuli for new doors of knowledge”.*

During all these years, since the publication of the first issue, many changes and advances^3^ have taken place in medicine and in general and digestive surgery; new exams emerged, improvement in pre and postoperative care and also in the surgeon-patient relationship^2^. Thus, both the ABCD and the CBCD have opened new doors for knowledge and have contributed to medical education in the country. Also, had its layout changed and modernized, making it more attractive and similar to the best international journals.

In 2009, ABCD was accepted and included in the list of national journals indexed in SciElo (Scientific Electronic Library Online) and started to be published in Portuguese and English online in PDF and HTML, in addition to the printed edition in Portuguese, thus having more visibility, appreciation and representing another leap in quality. In 2010, also through an agreement with other specialty societies, it became the official scientific organ of: Brazilian Association of Gastric Cancer, Brazilian Chapter of the International Hepato-Pancreato-Biliary Association, Pancreatic Disease Study Group, until 2017 to the Brazilian Society of Bariatric and Metabolic Surgery and the Brazilian Society of Minimally Invasive and Robotic Surgery (2014-2017).

This intersociety integration was awakened in me by Richard M. Satava, Professor in the Department of Surgery at the University of Washington, Seattle, USA, exponent of technological medicine and NASA consultant for medical affairs in spacecraft, when speaking during a magnificent conference in Munich in 2000 - he stated that: ... “*Whoever wants to live the century that is beginning will have to be focused on the “Information Age” and “Integration” applying them in their actions, as they will guide the winners of this century.”* It was with this thinking that the north of the ABCD was directed, not only in computerization and the use of digital technology, but also in the integration with similar societies. With regard to its publications and, understanding that integration is the word of intelligence today, it sought to approach the aforementioned similar associations and encouraged them to join forces instead of competing in isolation in the dissemination of their researches, showing signs that with acceptance of this way of thinking we can get the master lever to catapult oneself into the future, with gallantry and great success.

From 2012, it was with great pride that the editors communicated to CBCD members and all researchers in the great field of gastroenterology involving the aforementioned areas that the ABCD was included in the MEDLINE/PUBMED database, thus being placed among the best in the world^4^ (Figure 4).

The editing process of a scientific journal in medicine - although very little known by those who do not work in it - is extremely complex and laborious, and is permanently audited by the indexing databases in order to maintain its international visibility.

ABCD has had a brilliant career until today, which started from articles taken from the master’s/doctoral theses filed in the library of the Digestive Surgery Department of the University of São Paulo/Hospital de Clínicas, provided by Prof. Gama Rodrigues, and taken by me in suitcases (yes suitcases!) to Curitiba in order to be “photocopied” parts that, using them, were manually formatted for articles. At the time, there were not enough articles for the three-monthly editions. Today, we have approximately 300 submissions annually! Starting with indexing in SciELO in 2010 - after an incredible 11 years of trial and error! - the journal started to be not only printed as it had been until then, but with three editions: printed in Portuguese, online in Portuguese and online in English. What would they serve? The printed would continue like this since the birth of ABCD and would be distributed to all CBCD members in return for their association with the College and, also, sent to university libraries. Online in Portuguese would serve to publicize what we do in the country to Brazilians with digital access (let’s not think that everyone reads English). Online in English would serve for the journal’s internationalization, which is necessary to maintain the indexes we have, and which is the most important qualitative indicator for maintenance in the indexing databases.

**FIGURE 4:**
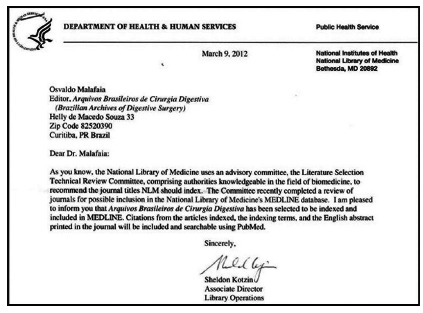
Announcement from the National Library of Medicine of the inclusion of the ABCD in the Medline/PubMed platform in 2012

In this way, and at this moment, it is easier to think of something that will further help the journal’s future. From this past many good things have happened. Speaking not of difficulties, but of achievements, we are today with very high academic recognition, nationally and internationally.

 The national one is shown by the submission of very good level works coming mainly from *stricto sensu* graduate programs in recent years. Renowned Brazilian authors have also frequently honored us, enhancing the merit of our journal, which is officially confirmed by its impact value currently provided: 1.797 (Figure 5). Magnificent!

**FIGURE 5:**
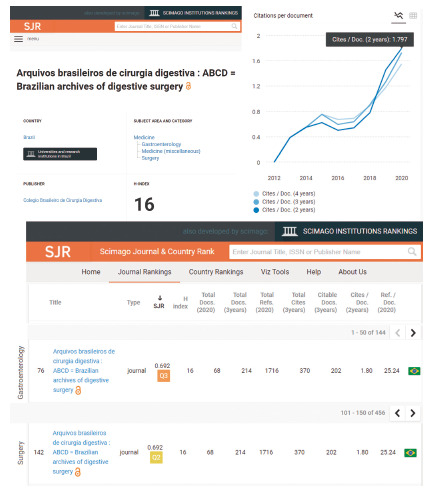
ScimagoJR impact factor and 76th place in gastroenterology and 142th in surgery

Its international penetration can be measured by the internationalization index registered in SciElo, around 30%, that is, 1/3 of our publications have total or partial origin from abroad. Today we also have all the most important indexes (Medline/Pubmed, PubMed Central, Scopus/Scimago, Web of Science - Emerging Sources Citation Index (ESCI), SciELO, Google Scholar, LILACS e DOAJ) that reassure us about the worldwide visibility of what we publish. It is always good to remember that the internationalization of any journal is no longer measured by the territoriality of a country, but by the virtual visibility that the journal’s digital media offer, regardless of its country of origin. Also, in relation to the international scenary, ABCD occupies 74^th^/144 and 142^th^/456 place among surgical journals worldwide, that is, of the 456 surgical journals indexed in Surgery/Scimago/Scopus, it is in the 2^nd^ quartile worldwide, among the best! Q2! (Figure 5). Of course, it will be very difficult for us to go much higher as we have already entered the territory occupied by the most renowned international journals, which are very strong. But, thinking smaller, we occupy the first place among surgical journals in Latin America (Figure 6 and access https://www.scimagojr.com/journalsearch.php?q=21100229216&tip=sid&clean=0). Beautiful!

**FIGURE 6:**
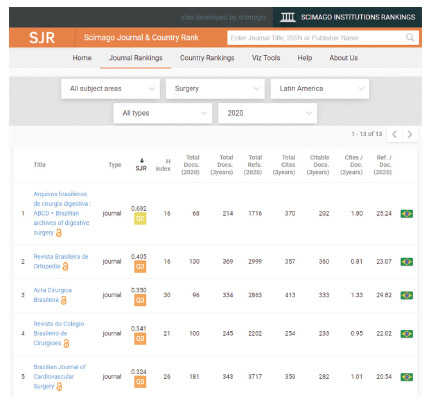
First place among surgery journals in Latin America

But none of this diminishes the need to move towards the ABCD. Suggestions are always welcome! And the most recent one was the use of Altmetrics to publicize what is done in the academy for social media. Focused mainly on Facebook, Tweeter and Mendeley, and describing what we do in less formal words coming from the academy, the advances and benefits of science for the population that use digital media to update themselves go viral in amazing numbers. It’s very interesting and it’s the modernity that all the big journals are using lately. For this, three small inserts were created: Image, Central Message and Perspectives. They can be viewed, and their content perceived, with a PDF download of any article from the last two years of ABCD (Figure 6). Modernly, it is required that with these and other altmetric measures the impact on society of what is produced in the academy can be measured.

**FIGURE 7:**
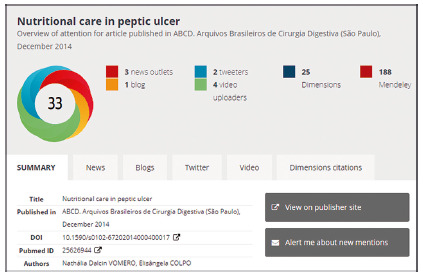
Example of altmetry with the various social media sources in which the article was cited

**FIGURE 8:**
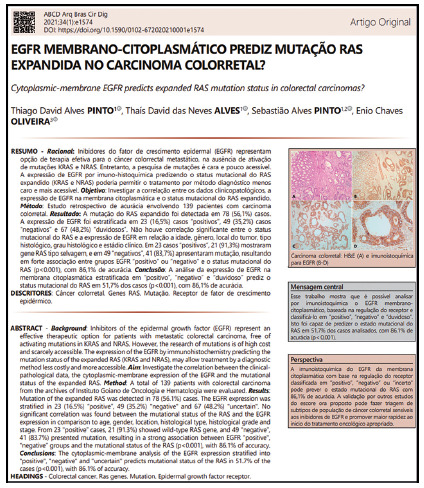
Image, Central message and Perspective

 But, for this to happen, it is necessary that the basic knowledge about what we are going to discuss to improve our journal can be measurable. If not, personal opinions are offered, which are often taken out of context.

The feeling at the end of my work in front of the ABCD is one of great satisfaction and of the duty fulfilled towards Brazilian surgeons, towards the members of the CBCD and towards the medical society in general. A journal that published over 1600 articles in its history and, of these, 1321 in the period I was Editor-in-Chief (2001/2021) is to be respected! All manuscripts underwent a detailed and thorough review by the editors and peers (peer-review), both with regard to their ethical and scientific content, as well as their writing in Portuguese and English (including comma review!), as well as its results and conclusions, its teachings and its final message. Certainly, the ABCD has reached its maturity. It is interesting to note that each article published has an average of 3000 words; if we multiply the number of articles during my editorial period it will mean a 3.9 million words in each of the two languages, that had to be analyzed for merit, spelling and their position in the linguistics of the text! Reinforcing: in Portuguese and in English, that is, 7,8 milion words! This huge number might be equivalent to an encyclopedia! We understand that our journal can have even greater national and international visibility and accreditation, if the better papers - which go to external journals - be concentrated on ABCD. It is the medical society as a whole that needs to collaborate. ABCD has done its part. Now!... Brazilians need to put aside other journals from outside Brazil - often with even less impact than ours - and publish on ABCD. I repeat: It is no longer the country where it is published that matters, as the internationalization of a journal is measured by the visibility contained in the “Information Age” (global indexing bases, interactivity with readers through videos, QRCode, social networks, and other digital means). With this thinking and the concentration of intellectual production in Brazil, showing what is produced here, this is how authors will be more valued and will also directly and indirectly increase the global number of national publications, raising our place in the world ranking of intellectual production measured by the indexing platforms. Let’s no longer impoverish our journals by sending “outside” (and sometimes saying it with pride!) what we think is best. ABCD is giving, with its demonstration of integration and strength, an opportunity to make our country more respectable and scientifically respectable, as it already is in other activities of knowledge and human development.

On this occasion, I have to thank the authors and co-authors who believed and sent their articles to ABCD over these 20 years, thus contributing to the dissemination of this fantastic and important amount of quality knowledge that we have, and who believed in the Journal’s progress. I have to thank the *stricto sensu* graduate programs, recommended by CAPES, who sent their theses for publication and all the editors, reviewers and collaborators who directly or indirectly contributed to the Journal’s permanence.

Finally, I believe that it is necessary to dream to crave and grow with victories and successes; but, for that, it takes a lot of dedication, detachment and teamwork! I leave the position of Editor-in-Chief of ABCD with great joy in my heart and soul, for delivering to CBCD a diamond of great carat - therefore of great value - and with exquisite cut, which reflects the brilliance it has today globally and in throughout Latin America.

I must thank all 13 ex-presidents for whom I served as Editor-in-Chief of ABCD. Many thanks to you for the unstinting trust, respect and support that I have been awarded in these 20 years. It is my deep desire that ABCD will continue to shine and increase its value, as we need it to have a better Brazil on the world scientific scenery.

I wish, from the deepest point of my heart and soul, success with the new guidelines to be drawn up and implemented for the continuity of the ABCD.

## References

[1] Pinotti HW (1986). Patient-surgeon relationship. ABCD Arq Bras Cir Dig.

[2] Andreollo NA, Cecconello I, Kruel CDP, Malafaia O (2011). 25 years of ABCD - surgeon-patient relationship in the past and present. ABCD Arq Bras Cir Dig.

[3] Andreollo NA, Malafaia O (2011). Silver jubilee of the ABCD. ABCD Arq Bras Cir Dig.

[4] Kruel C, Malafaia O (2012). ABCD included in Medline/Pubmed. ABCD Arq Bras Cir Dig.

